# Cardiac Amyloidosis and its New Clinical Phenotype: Heart Failure with Preserved Ejection Fraction

**DOI:** 10.5935/abc.20170079

**Published:** 2017-07

**Authors:** Evandro Tinoco Mesquita, Antonio José Lagoeiro Jorge, Celso Vale Souza Junior, Thais Ribeiro de Andrade

**Affiliations:** Universidade Federal Fluminense (UFF), Niterói, RJ - Brazil

**Keywords:** Amyloidosis, Heart Failure, Diastolic, Stroke Volume, Cardiomyopathy, Restrictive, Risk Factors

## Abstract

Heart failure with preserved ejection fraction (HFpEF) is now an emerging cardiovascular epidemic, being identified as the main phenotype observed in clinical practice. It is more associated with female gender, advanced age and comorbidities such as hypertension, diabetes, obesity and chronic kidney disease. Amyloidosis is a clinical disorder characterized by the deposition of aggregates of insoluble fibrils originating from proteins that exhibit anomalous folding. Recently, pictures of senile amyloidosis have been described in patients with HFpEF, demonstrating the need for clinical cardiologists to investigate this etiology in suspect cases. The clinical suspicion of amyloidosis should be increased in cases of HFPS where the cardio imaging methods are compatible with infiltrative cardiomyopathy. Advances in cardio imaging methods combined with the possibility of performing genetic tests and identification of the type of amyloid material allow the diagnosis to be made. The management of the diagnosed patients can be done in partnership with centers specialized in the study of amyloidosis, which, together with the new technologies, investigate the possibility of organ or bone marrow transplantation and also the involvement of patients in clinical studies that evaluate the action of the new emerging drugs.

## Introduction

Heart failure (HF) with preserved ejectionfFraction (HFpEF) is now an emerging cardiovascular epidemic, being identified as the main phenotype observed in clinical practice in different countries, such as the United States, United Kingdom, Portugal and Brazil. It is more associated with female gender, advanced age, and comorbidities such as hypertension, diabetes, obesity, and chronic kidney disease.^1-6^ Recently, a picture of senile amyloidosis has been described in patients with HFpEF, demonstrating the need for clinical cardiologists to investigate this etiology. Advances in cardio imaging methods combined with the possibility of performing genetic tests and identification of the type of amyloid material allow for greater ease in the diagnostic process in view of the clinical suspicion of the disease.^7-15^

Amyloidosis is a clinical disorder resulting from the deposition of insoluble fibril aggregates originated from proteins that have anomalous folding. These proteins, mostly initially soluble and with alpha helix configuration, take the pleated beta form through the abnormal folding phenomenon, with tissue precipitation in the form of amyloid fibrillar aggregates. These aggregates have the characteristic of staining congo red, acquiring a shade described as "apple-green" in polarized light. Through the alteration of the affected organ, it determines numerous dysfunctions of irreversible, progressive and indolent course, as observed in cardiac amyloidosis.^7,10,16-23^

Cardiac involvement may lead to the development of a restrictive HF model. Deposits in the myocardium and blood vessels cause diastolic, systolic dysfunction, ischemia and arrhythmias, and late diagnosis is the main cause of the reduction of survival of these patients.^7,10,16-23^

The diagnosis of amyloidosis presents important non-invasive advances in characterizing its presence and its type. In the past, the diagnosis was centered on the endomyocardial biopsy stained by congo red. More recently, new techniques such as doppler echocardiography with analysis of myocardial strain, myocardial scintigraphy with radioisotopes such as Tc99m bound to pyrophosphate or 2,3-dicarboxypropane-1,1-diphosphonate (DPD) and magnetic resonance imaging of blood tests for genotyping evaluation have promoted important advances in this area.^11,12^

New treatments directed toward specific disease targets have already been incorporated into clinical practice and others are still being tested, gradually improving patients' survival and quality of life.^24,25^

**Figure 1:**
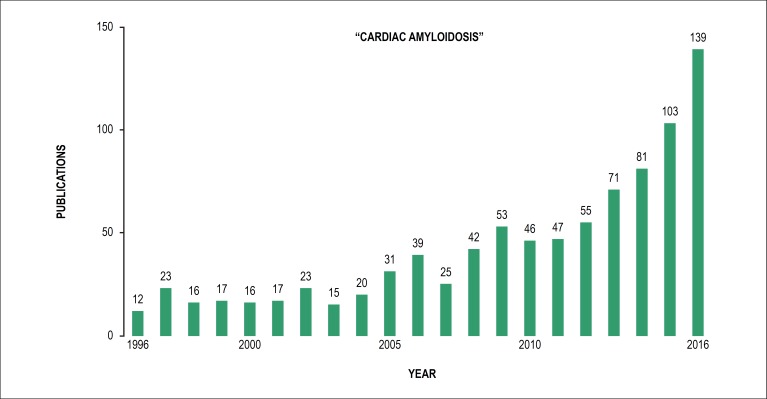
Search with the term Cardiac Amyloidosis from 1996 to 2016 showing the growth of articles related to the topic in the last ten years. (Medline)

According to data obtained in MedLine, publications on the cardiac amyloidosis framework date back to 1948, totaling over 1000 articles indexed in several languages. Over the last five years, there has been a continuous increase in the number of studies evaluating the various aspects of the disease, especially with regard to innovations in diagnostic methods and new therapies (Figure 1). This trend is confirmed by the fact that the material produced in the last five years represents a third of the total published so far.^26^

The increase in the number of studies on the disease provides sufficient evidence for physicians to increase their clinical suspicion of cardiac amyloidosis, especially of the senile type, in cases of HFpEF, and referral of these patients to specialized centers is recommended. At these sites, invasive and non-invasive diagnostic methods allow for a wide assessment, including genetic testing, multidisciplinary teaming, and access to new drugs.

In this present review we will discuss the recent advances in the etiology and pathophysiology of cardiac amyloidosis, especially the senile form, in a systematized way for the evaluation of individuals suspected of amyloidosis in the context of HFpEF and the emerging therapies currently available in clinical practice.

### Classification and etiopathogenesis of amyloidosis

In the face of the complexity associated with the disease and its multiplicity of presentations, specific nomenclatures and classifications were established related to the predisposing condition and to the type of amyloid fibril deposited in the tissue. In general, amyloidosis can be classified as primary, secondary, related to dialysis and associated with transthyretin.^27-30^

It is classified as primary amyloidosis (AL) when it is defined by the production of amyloid protein composed of light chain immunoglobulins (kappa and lambda), synthesized under clinical conditions that present plasmacyclic dyscrasias, such as multiple myeloma and, less frequently, Waldenström's macroglobulinemia and non-Hodgkin's lymphoma. Classically, AL is a systemic disease that predominates in an older population and in male individuals.^27,31^

The clinical picture varies directly with the organ of predominance of amyloid deposition and its degree of functional impairment. The two most commonly affected organs are the kidney and heart, accounting for 60-80% of patients in most studies. Kidney involvement manifests as nephrotic syndrome or asymptomatic proteinuria. Cardiac involvement is related to the development of a HFpSC, in addition to the possible involvement of the heart conduction system and its corresponding complications. Autonomic neuropathy, or sensitivomotor peripheral neuropathy, may be present in up to 20% of patients.^27,32-34^

The accumulation of the amyloid material in the liver is frequent and comes along with isolated hepatomegaly or even hepatosplenomegaly, and may present a pattern of elevation of liver enzymes, compatible with cholestasis. Muscular infiltration may occur with pseudohypertrophy, as in classical macroglossia, as well as arthropathy due to deposits in the joints. Periorbital purpura (raccoon's eyes), despite being an uncommon finding, is strongly characteristic of the AL form. Hemorrhagic diathesis is an important condition that may be present and reports as possible causal links the connection between amyloid material's with coagulation factor X, the reduced synthesis of coagulation factors in the presence of a compromised liver and a possible acquired von Willebrand's disease.^27,32-34^

Secondary amyloidosis (AA) is identified in chronic inflammatory clinical conditions such as rheumatoid arthritis, psoriasis and, more recently, autoinflammatory diseases such as inflammatory bowel disease, Mediterranean family fever, and Muckle-Wells syndrome. Fibrils are composed of the amyloid A protein and are produced by the liver during the acute phase of inflammatory diseases. This protein originally has the function of increasing the affinity of high density lipoprotein (HDL) by macrophages and adipocytes, as well as mediating the chemoattraction and induction of the synthesis of proinflammatory cytokines. The chronic inflammatory picture increases its synthesis and, due to incorrect processing with cleavage and erroneous folding, results in its pathogenic form. The kidneys are involved in approximately 80% of patients, being the organ most affected by AA. However, there are also reports of cardiac involvement.^27,35-37^

Dialysis-related amyloidosis occurs as a function of the deposition of fibrils originating from beta-2 microglobulin proteins, which accumulate at increasing levels in patients with advanced renal disease and who undergo long-term dialysis. In this form, in particular, the predominant condition is osteoarticular involvement, such as carpal tunnel syndrome and rotator cuff involvement.^38,39^

Cardiac amyloidosis associated with transthyretin (TTR) is the second form of amyloidosis with a higher prevalence of cardiac involvement, and may be divided into hereditary and senile forms. The precursor protein is predominantly synthesized in the liver and plays a role as a carrier of retinol and thyroxine.^10,14-18^

In the senile form, we identifie tissue deposition of the TTR wild form, especially in the myocardium, and a clinical picture of HF is observed. The association with carpal tunnel syndrome has been described, whereas renal involvement is a rare finding. It has been observed in necropsy studies that deposition of this amyloid material in the heart is a frequent finding, especially in previously asymptomatic patients.^22^ Data from the Mayo Clinic group indicate that the prevalence of this form among patients with amyloidosis is approximately 8,5%, with a mean age of 77 years and the male sex representing 82% of the affected individuals. It has been found that it rarely occurs in patients below 70 years of age. Observational study, observed that patients usually have a slow progression and a survival, after the diagnosis, of approximately 43 months, compared to the 26.6 months of the mutant form.^18,40-42^

The hereditary form, unlike senile, affects patients in different age groups, but predominates a mean age lower than that found in patients with the senile form. The coding of TTR occurs on chromosome 18 and more than 70 mutations associated with this protein have been identified. In view of the suspicion of an amyloidosis by TTR, the sequencing of this protein from tissue or blood sample should be performed for the diagnosis and identification of a possible specific mutation that allows us to define the prognostic course of the patient and guide the investigation of the relatives. The Val122Ile mutation is more associated with the elderly and is predominantly male, presenting in 90% of the cases a clinical manifestation of a cardiomyopathy.^7,10,14,15^

The most prevalent mutation in the world population is Val30Met, which presents marked neurological involvement, allied to a late cardiac involvement and is related to Corino de Andrade's disease, also known as familial amyloid polyneurophaty (FAP), which occurs along with sensory-motor peripheral polyneuropathy. Manifests, especially, at the age of 20 years, characterized by paresthesias, motor and autonomic disorders, besides studying with cardiac and renal impairment in the late phase of the disease. This condition has been identified as a genetic disease associated with the TTR mutation.^7,10,14,22^

### Clinical presentations

Amyloidosis cardiomyopathy is classically described as a directt HF model, often occuring together with ascites, predating lower limb edema and allying with hepatomegaly on physical examination. Unlike cardiac dysfunction with increased filling pressures, pulmonary edema is an infrequent condition in amyloidosis cardiomyopathy.^7,8,10,14^

A rarer phenotype is the involvement of the interventricular septum with deposit of the amyloid material promoting the disproportionate thickening of the region, mimicking a hypertrophic cardiomyopathy. This presentation constitutes what is called a phenocopia, that is, a clinical condition that presents itself through manifestations typical of a disease of well-defined genetic origin.^21,24,41^

The report of syncope, due to autonomic nervous system involvement by amyloidosis, is a common finding in these patients and their presence in relation to physical exertion is associated with a worse prognosis, presenting a high mortality in three months, often due to sudden death.^21,41^

Ventricular arrhythmias are uncommon causes of syncope in this population. This is justified by the fact that the myocardium infiltrated by the amyloid material is more susceptible to episodes of hypoperfusion. Diseases of the conduction system may be present in the different forms of amyloidosis. However, they are more frequently found in the form associated with TTR, both senile and hereditary. Syncope in patients with cardiac amyloidosis is mainly associated with hypotension due to dysautonomia and bradyarrhythmias and less related to ventricular arrhythmias. Malignant ventricular arrhythmia, when present, is a common cause of death in patients with cardiac amyloidosis, and these patients are strong candidates for implantation of cardio-defibrillators.^24^

Involvement of the pericardium may occur in some cases, resulting in pericardial effusion which, most of the time, does not develop cardiac tamponade. Due to the alterations of cardiac amyloidosis itself, this condition may be masked and not have echocardiographic signs such as atrial and right ventricular compression.^24,41^

The accumulation of the material in the atrium promotes its electromechanical dysfunction and, consequently, considerably increases the risk of intracavitary thrombus formation. This process is evident, especially in patients with AL amyloidosis type and is an independent factor of atrial fibrillation, and when both factors are present, the risk of thromboembolism is extremely high. Therefore, the use of anticoagulants should be considered in these individuals.^24,41^

### Cardiac amyloidosis and its new clinical phenotype

A new insight into HFpEF becomes critical, given its increasing relevance as the most prevalent clinical phenotype of HF in the world population. This data is present in our country, as evidenced by the DIGITALIS study, which investigated the prevalence of HF and its phenotypes in primary care in the city of Niterói. According to this study, among the population with HF, 59% had the HFpEF phenotype.^5^

Data from a specialized center in amyloidosis in Brazil point to a high prevalence of myocardial involvement in patients with amyloid polyneuropathy from abnormalities on the electrocardiogram (ECG).^43^

Despite numerous studies about this clinical condition, much is unknown about its etiophysiopathogeny, which has caused negative results in the studies of treatment of HFpEF. This is hampered in particular by the numerous phenocopies that mimic their presentation.^6,44^

In this scenario, it is worth highlighting the possibility that a portion of the patients with HFpEF actually present a cardiac amyloidosis. This is confirmed by recent studies with patients with a diagnosis of HFpEF, in the absence of arterial hypertension or diabetes, through the new cardio-imaging modalities, present accumulation of amyloid material in the myocardium. Next to this is the fact that amyloid material has been identified in necropsy studies of patients with HF.^7,8,15,45^

In order to facilitate the identification of a suspected cardiac amyloidosis, regardless of the type of fibril deposited, we must consider some clinical evidence and complementary tests as presented in Table 1.^7^

**Table 1:** Clinical criteria and complementary tests in the investigation of cardiac amyloidosis

Categories	Criteria
History	Age of onset of HFpEF > 60 years
	Family history of unexplained HF at age 60
	Peripheral polyneuropathy
	Carpal tunnel syndrome
	Blood dyscrasia
Physical exam	Orthostatic hypotension
	Macroglossia
	Unexplained skin lesion
Medicines	Beta-blocker intolerance
	Vasodilator intolerance
ECG	Dissociation between low voltage ECG with ECHO hypertrophy
	Atrial Fibrillation / Flutter
	Bloqueio atrioventricular
	Pseudoinfarction pattern
ECHO	Unexplained ventricular hypertrophy
	Increased interatrial septum thickness
	Increased myocardial granulation
	Biatrial increase
	Restrictive filling pattern (Increased E/A and E/E ratio)
	Preservation of longitudinal strain
	Pericardial effusion

HfpEF: Heart failure with preserved ejection fraction; ECHO: doppler echocardiography; ECG: electrocardiogram.

**Figure 2:**
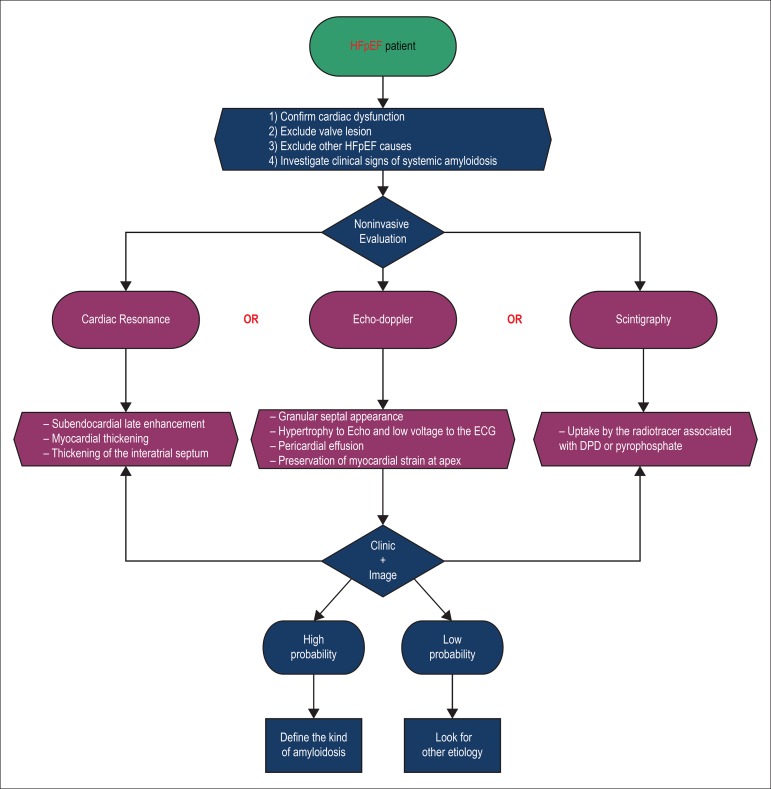
Flowchart for the evaluation of patients with HFpEF and suspicion of amyloidosis. HfpEF: heart failure with preserved ejection fraction; Echo: doppler echocardiography; ECG: electrocardiogram; DPD: 2,3-dicarboxypropane-1,1-diphosphonate.

**Figure 3:**
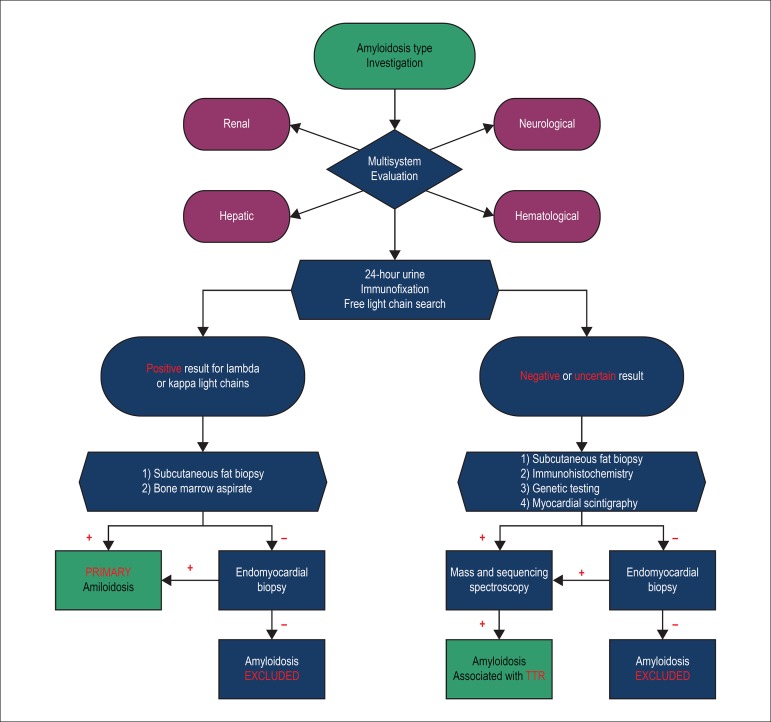
Flowchart for investigation of the type of amyloidosis. TTR: transthyretin.

In this scenario where the most common etiologies applicable to the HFpEF are not confirmed, a cardiac amyloidosis scenario should be suspected. Therefore, we propose a guideline flow chart for the management of these patients (FIGURES 2 and 3).^18,22^

### Diagnostic approach

Cardiac amyloidosis presents an indolent course and the diagnosis is made, often late, thus contributing to a worse prognosis. In several situations, cardiac involvement causes great morbidity to the patient, although the diagnosis is often not suspected, even in conditions where there is the characterization of restrictive cardiopathy.^7^

In clinical history, cardiac amyloidosis may be suspected in patients 50 years of age or older who have signs and symptoms of HF, with an ejection fraction of the left ventricle greater than or equal to 50% and that do not show improvement of the symptoms with the treatment. Extracardiac manifestations in the patient's history should be considered as guiding principles for the diagnosis of HF by amyloidosis, such as peripheral neuropathy and carpal tunnel syndrome, especially recurrent and bilateral. Signs and symptoms such as orthostatic hypotension, macroglossia, muscular consumption of the tenar and hypothenar region, haematomas of unknown origin and cardiac electrical conduction disturbances may also be present.^7,29^

The ECG is an easily accessible examination that may offer changes that raise the suspicion of amyloidosis in the presence of a CHF. The finding of a low voltage QRS complex, electrical axis deviations and branch block can be found. Arrhythmias are frequent, especially atrial fibrillation, which is related to amyloid infiltration of the atrium, and complex ventricular arrhythmias.^14,22^

ECHO is an important method of diagnostic investigation and the presence of significant left ventricular hypertrophy associated with ECG with low-voltage QRS may lead to suspicion of cardiac amyloidosis. Another criterion for suspicion of cardiac amyloidosis is thickening of the left ventricular wall above 12 mm in the absence of a history of systemic arterial hypertension. Other findings that may be present in ECHO are the biatrial increase with normal-sized ventricles, pericardial effusion, and evidence of diastolic dysfunction due to the pattern of restive cardiomyopathy.^9,14,22^

Measurement of the thickness of the interventricular septum may suggest the type of amyloidosis present in the patient, and is often greater in cases of amyloidosis by TTR than the AL form, and may in many cases be greater than 20 mm. However, the separation between these two forms on a clinical basis is not always possible.^9,14,22^

In some patients with cardiac amyloidosis, we can observe the clinical phenotype similar to obstructive hypertrophic cardiomyopathy due to the presence of the dynamic pressure gradient that is related to an additional "narrowing" of the left ventricular outflow tract. More recently, longitudinal systolic strain has been used for the diagnosis of systolic dysfunction in patients with cardiac amyloidosis, and may show the preservation of the heart tip in relation to other walls. In the presence of ECG and ECHO alterations we should resort to complementary tests. In a first step in the elucidation of a case of cardiac amyloidosis, we investigated the renal function status of this patient through the nitrogenous excoriations and, in particular, the quantification of protein loss and its type, made through the collection of 24h urine with dosage proteinuria and urinary protein electrophoresis. This stage in the investigation allows the identification of the primary form of amyloidosis and consists in the identification of the light chains that are in high titers in these patients. Immunofixation, when associated, allows an increase in the diagnostic accuracy of this type of presentation.^9,14,22,31,34^

The abdominal fat aspirate for histopathological study is a more accessible alternative, since it is a simple diagnostic procedure, easy to perform, safe and that presents good sensitivity, but having less accuracy in the form associated with TTR. However, when a negative result is obtained with abdominal fat aspirate, right ventricular endomyocardial biopsy may be essential for the diagnosis of cardiac amyloidosis. Through this method, the amyloid protein is stained by congo-red. Other tissues can also be evaluated as the rectum, gums, bone marrow, kidney, among others. The histochemical study of tissue samples is important in order to distinguish between the hereditary, senile, systemic and primary forms, due to differences in treatment and prognosis.^14,22,29^

Magnetic resonance imaging appears as another alternative for the diagnosis of cardiac amyloidosis, with a sensitivity of 87% and specificity of 96% for the form associated with TTR. Through this examination, it is possible to identify the myocardial and atrial septal thickening, signs of diastolic dysfunction, and the typical pattern of late subendocardial enhancement in the left ventricle, which may also affect all cardiac chambers.^11,12,22,29^

Molecular imaging has also revolutionized diagnosis. The non-invasive method can be used from the use of the Tc99m radiotracer, which binds to TTR but not to the light chain derivatives, being an effective method of evaluating the mutant or wild forms of cardiac amyloidosis associated with TTR. Positron emission tomography in conjunction with the C-RiB plotter may be a new strategy to be used in the diagnosis of these patients.^14,22^

Bone marrow biopsy with immunohistochemical staining or flow cytometry analysis is critical in patients in whom the type of AL-amyloidosis has been identified. This will demonstrate a clonal population of plasma cells, which are producing defective light chains of the antibody. If these tests are negative, we should investigate the hereditary forms of the disease.^29,33^

There are new studies involving omic sciences that aim to increase the diagnostic accuracy of amyloidosis. Proteomics involves the study of all the protein expression of a cell in different conditions, being its study complementary to the genome, identifying any protein, with or without genetic mutations. The main technique employed is laser microdissection followed by mass spectrometry (LMD-MS), through which positive samples in the congo-red color are dissected and decomposed into smaller components called peptides.^25,28^

### Treatment of cardiac amyloidosis

Treatment of cardiac amyloidosis is best performed in specialized centers of the disease. Treatment requires two approaches: control of heart-related complications due to amyloid deposition and treatment of the underlying disease to prevent new amyloid formations.

Treatment of cardiac amyloidosis aims to improve the signs and symptoms of HF. The use of low-dose diuretics improves symptoms related to congestion, while the use of the combination of beta-blockers and angiotensin-converting enzyme inhibitors has its undefined benefit in amyloidosis.^16,18,21,22,24^

The use of digitalis has no benefits in this group of patients, since the myocardium in dysfunction by the amyloid material is more susceptible to toxic effects, which predisposes to the occurrence of arrhythmias.^16,18,21,22,24^

The use of anticoagulants should be considered in case of atrial fibrillation and in the detection of intracavitary thrombi.^18,21,22,41^

### Amyloidosis of AL form

Overall survival is approximately four years after diagnosis and has improved over the past three decades. AL-amyloidosis is often the result of a clonal increase of plasma cells in the bone marrow and thus therapy with cytotoxic chemotherapeutics may be effective. The performance of the hematologist in the staging process and definition of the therapeutic strategy in this scenario is fundamental.^21,31,33,34^

Patients who present a hematological response to treatment have symptomatic improvement and cardiac biomarkers, and can occur along with amyloid deposition, which is already evident in the first three months, confirming a better prognosis in these cases.^19,21,31,33,34^

Dexamethasone-associated melphalan therapy (MelDex) in patients ineligible for autologous stem cell transplantation had a response rate around 70%, which was worse in cases with advanced cardiac involvement. In a recent randomized clinical trial comparing MelDex with high doses of melphalan followed by stem cell transplantation showed a better survival rate in patients who took MelDex.^32,46-48^

Bortezomib has been shown to be an effective drug when combined with cyclophosphamide and dexamethasone, with a significant hematological response (71%) after two months of use. According to Mayo Clinic group, in the case of three patients initially ineligible for stem cell therapy, the use of bortezomib made the procedure possible. Stem cell transplantation is used in 25% of patients with cardiac amyloidosis. Following the procedure episodes of supraventricular tachycardia may occur, with mortality of 11% in these individuals. The positive hematological response in patients submitted to stem cell therapy is approximately 56%.^16,19,27,32,33^

A study evaluating the use of bortezomid, dexamethasone and alkylating agents (BDEX + AA) in 106 patients with symptomatic HF due to AL cardiac amyloidosis showed an improvement in survival after adjustment of clinical variables. (Hr: 0.209, 95% CI: 0.069 to 0.636, p = 0.006).^32,49^

### Form associated with TTR

Tafamidis appears as an important option for the treatment of amyloidosis, acting as a kinetic stabilizer of the TTR tetramer. The interaction of molecules at certain TTR binding sites promotes stability to the protein in its tetrameric state, markedly decreasing its dissociation and, consequently, the amyloidogenesis. Tafamidis has the ability to selectively bind to one of the thyroxine sites in the TTR, promoting the kinetic stabilization of the tetramer.^7,22,24,32,50-54^

In a multicenter, randomized, double-blind, placebo-controlled study, the safety and efficacy of oral Tafamidis in patients with amyloidosis and involvement of the peripheral nervous system were demonstrated. Clinical trials show that this medication slows the progression of the disease, improves the function of small and large caliber nerve fibers and, consequently, reduces the functional loss of the affected systems, optimizing the quality of life of the patient. In another study, Tafamidis resulted in stabilization of transthyretin in 97% of patients with mild to moderate HF due to the wild type of cardiac amyloidosis.^7,22,24,32,50-54^

Another drug in the evaluation process for the cardiac amyloidosis associated with TTR is Diflunisal, a non-steroidal anti-inflammatory that can stabilize the tetramer, avoiding amyloidogenesis. One cohort evaluated the tolerance and effects promoted in 13 patients with cardiac amyloidosis by TTR, both mutant and wild type. No significant changes in cardiac structure and function were observed, as well as biomarkers.^32,55,56^

The use of doxycycline and tauroursodeoxycholic acid was carried out with a small number of patients, and a possible clinical improvement was identified. A new second-generation antisense therapy, ISIS-TTRrx, works by reducing the serum level of the TTR protein by suppressing the gene expression of its synthesis. In addition, a total of 28 studies are enrolled in the Clinical Trials database for interventions in patients with cardiac amyloidosis.^32,57,58^

An alternative treatment for some types of amyloidosis would be liver transplantation with the goal of replacing the mutated TTR gene that produces the majority of the circulating transthyretin by a gene found in a genetically normal donor organ. In this way, liver transplantation may be an alternative to slow the progression of the disease and prolong the patient's survival. However, chronic immunosuppression pertinent to transplantation leads to a high mortality rate in the first year, about 10%, and high morbidity. Transplantation does not prevent the extrahepatic synthesis of amyloid protein and thus does not delay the progression of the disease.^22,24,32^

## Conclusion

Cardiac amyloidosis inaugurates a new era of personalized cardiology, where precise diagnosis through techniques involving molecular genetic analysis, biomarkers and cardioimaging methods make it possible to classify the form of amyloidosis and define its clinical course and prognosis and, in the future, guide the therapeutics of these frames.

The clinical suspicion of amyloidosis should be increased in cases of HFPSE in which the methods of cardiac imaging are compatible with the restrictive cardiomyopathy or signs of dissociation between ECHO and ECG findings. The partnership with centers specialized in amyloidosis combined with new technologies are fundamental in the management of these patients through specialized treatments, including organ transplantation, or even involving patients in clinical studies that evaluate the action of new emerging drugs.
